# Bone wax migrates to the orbit in a patient with a frontal sinus abnormality: a case report

**DOI:** 10.1186/s12886-019-1037-x

**Published:** 2019-01-24

**Authors:** Yangbo Zhou, Minhong Li, Xin Wei, Xue Yang, Jialin Zhang, Xin Qi

**Affiliations:** 1 Department of Ophthalmology, the 2nd Xiangya Hospital, Central South University, Changsha, 410011 Hunan Province China; 2 Hunan Clinical Research Center of Ophthalmic Disease, Changsha, 410011 Hunan China; 3 Department of Neurosurgery, the 2nd Xiangya Hospital, Central South University, Changsha, 410011 Hunan China; 4 Department of Radiology, Hainan Fifth People’s hospital, Haikou, 570100 Hainan China

**Keywords:** Bone wax migration, Orbit, Ptosis, Proptosis, Craniotomy, Frontal sinus, Case report

## Abstract

**Background:**

Bone wax is the most widely used hemostatic bone sealant because of its availability, ease of use, immediate action, and minimal adverse effects. Several complications have been reported to be associated with the use of bone wax, such as infection, osteohypertrophy, pain, granuloma formation, allergic reaction, and thrombosis. Here, we present a rare complication, namely, bone wax migration, which developed after a craniotomy on a patient who had a frontal sinus abnormality.

**Case presentation:**

A 51-year-old woman complained of pain and swelling in her left eye accompanied by difficulty opening the left eyelid after undergoing a craniotomy. An examination revealed left eye proptosis with ptosis, eyelid swelling, and increases in intraorbital pressure and intraocular pressure (IOP). According to a CT and an MRI of the orbit, we found that the intraoperative bone wax had migrated to the orbit, thereby causing compression. We also found that the basal frontal sinus of the patient was congenitally defective, which may have induced the migration of the bone wax. Given that the patient recently underwent a craniotomy and given the risks associated with orbital surgery, she refused to undergo a surgery to remove the bone wax. Thus, the patient was administered mannitol intravenously daily, accompanied by topical Timolol, to reduce the intraorbital pressure and IOP. This treatment led to a gradual decrease in IOP and intraorbital pressure, and these parameters remained stable after treatment ended. During the 6-month follow-up, the best corrected visual acuity improved, and ptosis and restricted eye movements also improved significantly.

**Conclusions:**

We report a case of bone wax migration that developed after a craniotomy on a patient who had a congenital defect in the basal frontal sinus. Extra caution should be taken when using bone wax, and a comprehensive understanding of the patient’s intracranial anatomy is important for decreasing the incidence of bone wax migration. Additionally, when a patient presents with symptoms of ocular compression, bone wax migration should be considered in addition to typical radiological changes.

## Background

Bone wax is the most widely used hemostatic bone sealant because of its availability, ease of use, immediate action, and minimal adverse effects [1]. It is routinely used in neurosurgery, craniofacial surgery, orthopedics, thoracic surgery [2], and sometimes orbital surgery to reduce bleeding and to close the window from bone structures. Several complications have been reported to be associated with the use of bone wax, such as infection, osteohypertrophy, pain, granuloma formation, allergic reactions, and thrombosis [1, 3, 4]. Here, we present a rare complication of bone wax, namely, ocular compression resulting from bone wax migration, which developed after a craniotomy on a patient who had a frontal sinus abnormality.

## Case presentation

**Fig. 1:**
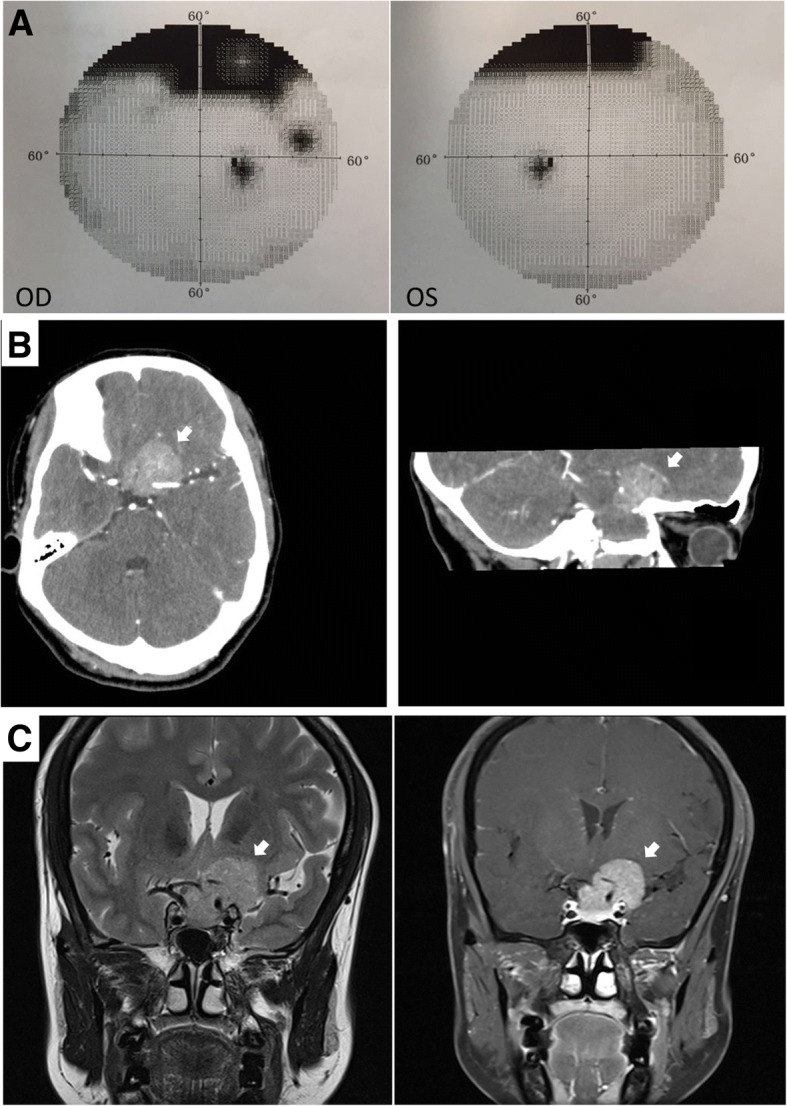
**a**: Visual field: the superior visual field was narrowed in both eyes, considering caused by drooping upper eyelids. **b**: Brain CT: showing a mass in sellar-suparsellar-parasellar region (white arrow). **c**:Brain MRI: showing an irregular soft tissue mass with uniform T1 and long T2 signal(white arrow), which was lobulated, with clear boundaries and compression on the left temporal lobe of the brain

A 51-year-old woman was referred because of a 20-year history of intermittent headaches and dizziness that had been accompanied by blurred vision in both eyes for 10 years. The patient did not have a family history of glaucoma. An ophthalmic examination of the patient revealed that although the best corrected visual acuity (BCVA) values were 0.8 and 0.5 in her eyes and that the intraocular pressure (IOP) was normal in both eyes, the superior visual field was narrowed in both eyes, likely due to drooping upper eyelids (Fig. 1-a). A neurological examination revealed that the muscle strength and muscle tension of the limbs were normal, and Babinski’s sign, Kernig’s sign, and Brudzinski’s sign were negative. A CT scan (Fig. 1-b) and an MRI (Fig. 1-c) of the sella revealed a mass in the sellar-suparsellar-parasellar region; this mass was likely to be a meningioma, which compressed the left optic nerve. We then performed a craniotomy using the anterior cranial base approach. During the operation, the frontal sinus apex was opened, and the tumor was found to be surrounding the left optic nerve, which is also close to the internal carotid artery and the oculomotor nerve. We removed as much of the tumor as possible, sealed the top of the frontal sinus with bone wax, and sutured the epidural.

**Fig. 2:**
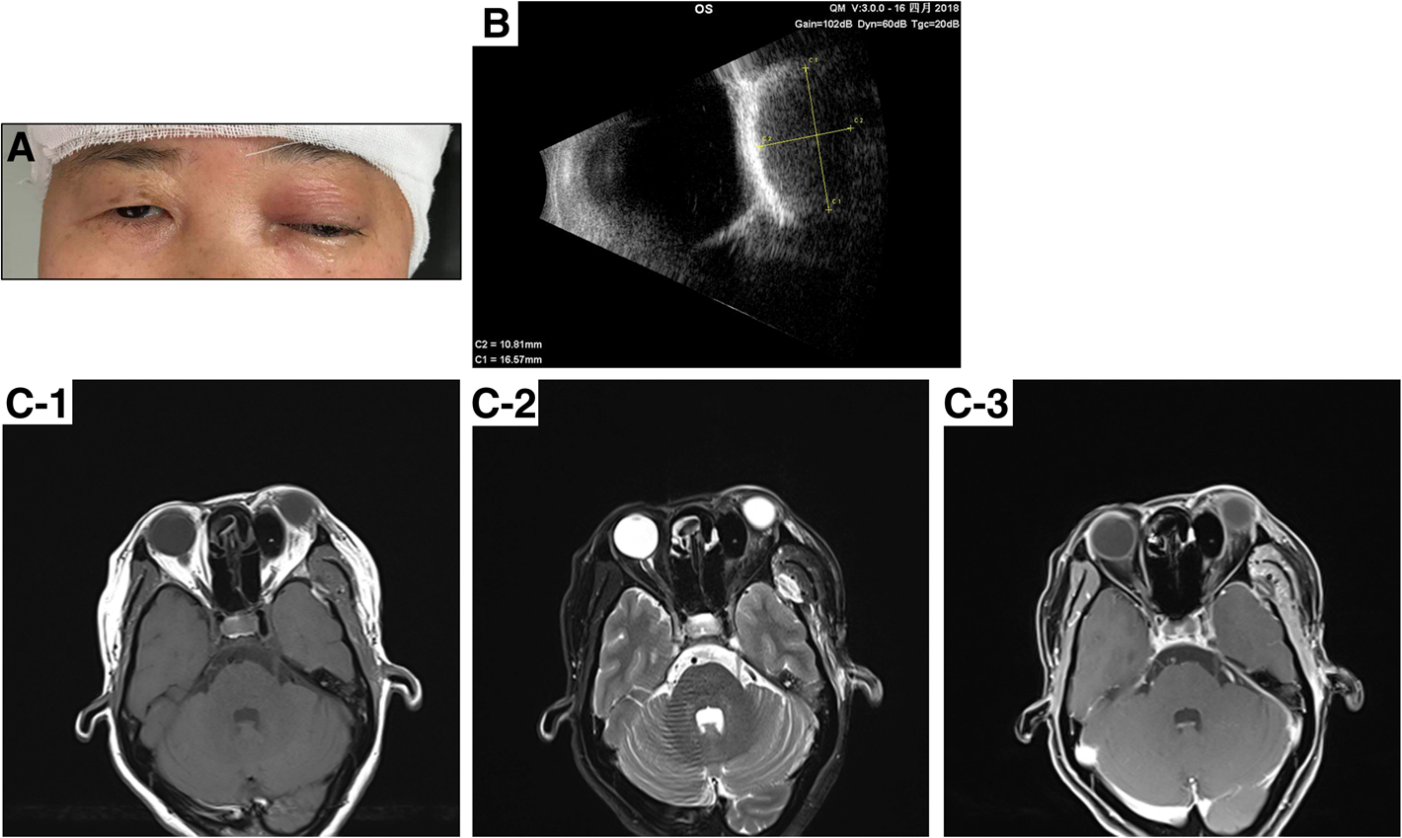
**a**: The left eye proptosis with ptosis, eyelid swelling; **b**: B-scan revealed that an oval mass with regular shape in the left orbit, size 10.81 mm X 16.57 mm approximately. **c**: Orbit MRI revealed that a low T1 and T2 signal mass with regular oval shape was detected in the upper nasal side of left orbit, without enhancement, the left optic nerve, superior rectus muscle and left eye ball were significantly compressed

The operation was successful. A postoperative CT showed that the lesion in the saddle area had been removed, and a small amount of blood and effusion accumulated under the dural membrane of the left frontotemporal region. On the third day after operation, the patient complained of swelling and pain in her left eye, accompanied by difficulty opening the left eyelid. An examination revealed proptosis with ptosis in the left eye, eyelid swelling, and increases in intraorbital pressure and IOP (Fig. 2-a). The left eye movement, especially upward movement, was limited, and the BCVA was limited to 0.1, yet the RAPD was negative. A B-scan (Fig. 2-b) and an MRI (Fig. 2-c) revealed a regularly shaped, hypodense, oval mass in the upper nasal side of the orbit; the left optic nerve, superior rectus muscle and left eyeball were significantly compressed, likely due to a foreign body, such as intraoperatively placed bone wax. Given the patient’s recent craniotomy and given the risks associated with orbital surgery, she refused to undergo a surgery to remove the bone wax. Thus, to reduce IOP, the patient was administered mannitol intravenously (125 ml q8h) daily, accompanied by Timolol topically. This treatment led to decreased IOP and intraorbital pressure, and the parameters remained stable after treatment. Three weeks after the craniotomy, the BCVA improved to 0.2, and the patient was discharged.

**Fig. 3:**
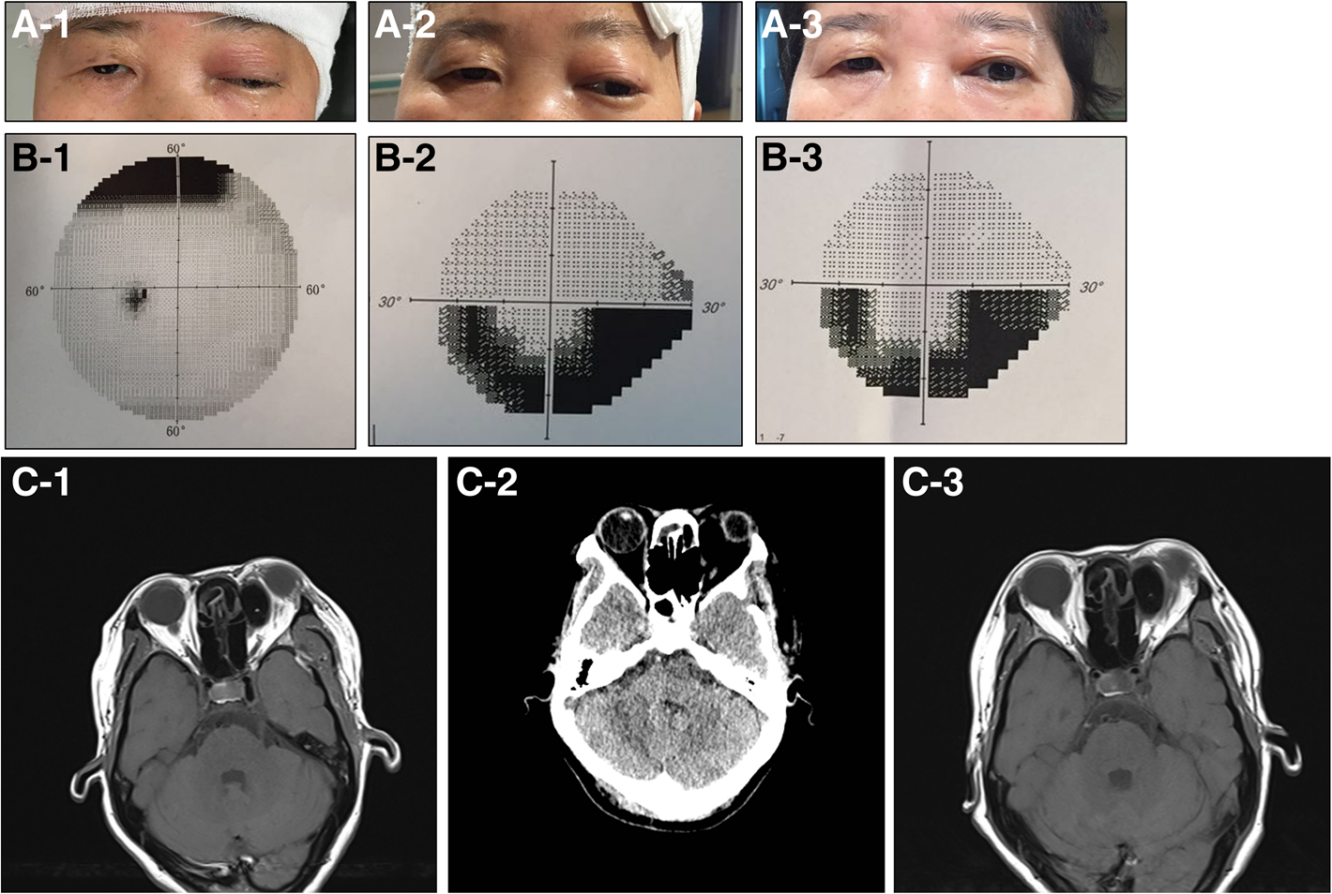
**a**: The ptosis and protosis of the left eye. A-1: POD 3; A-2: POD 1mo.; A-3: POD 6mo. **b**: The visual field of the left eye. B-1: POD 3; B-2: POD 1mo.; B-3: POD 6mo. **c**: MRI/CT of the orbit. C-1: POD 3; C-2: POD 1mo.; (CT); C-3: POD 6mo

At 1 month of follow-up, although the BCVA of the patient’s left eye improved to 0.6 and ptosis and restricted eye movements improved significantly, there was still proptosis with respect to the orbit. Ophthalmic examination revealed that the thicknesses of the retinal nerve fiber layer in the superior and temporal left eye were decreased with a narrowed inferior visual field. A B-scan showed that the retrobulbar mass was still not absorbed (Fig. 3). Removal of the bone wax seemed necessary for improving the patient’s ophthalmic and neurologic symptoms. However, the patient refused to undergo a surgery due to the associated risks. At a 6 month follow-up, the retrobulbar mass did not change significantly (Fig. 3), the ophthalmic symptoms of the patient remained stable, and the visual field of the left eye did not appear to improve.

## Discussion

Bone wax is a mixture of sterile beeswax (70%) and Vaseline (30%), which is routinely used in neurosurgery, craniofacial surgery, orthopedics, thoracic surgery [2], and sometimes orbital surgery to reduce bleeding and to seal bony window defects. Several complications have been reported to be associated with the use of bone wax, such as infection, osteohypertrophy, pain, granuloma formation, allergic reactions, and thrombosis [1, 3, 4]. Rare complications, such as ptosis and diplopia, were reported in a recent case of craniotomy [5]. Here, we presented a similar case of postoperative ptosis and proptosis as a complication of bone wax migration. In our case, intraoperative bone wax migrated to the orbit, thereby compressing and might damaging the superior rectus muscle, the levator palpebrae muscle, and the oculomotor nerve, which led to ptosis, proptosis, and eyeball movement limitation. Prolonged increased intraorbital pressure further resulted in visual field defection. During this situation, systemic dehydration and anti-inflammatory treatment could only partially release edema caused by early postoperative inflammatory response, and given that the bone wax is a nonabsorbable agent and remains in the body indefinitely, long-term residue could cause granuloma formation and lead to further damage; thus, performing orbital decompression to remove bone wax [6] seems to be the only and necessary procedure to ameliorate the ophthalmic and neurologic symptoms. However, the patient in our case refused further treatment due to the risks associated with surgery and thus suffered an irreversible loss of vision.

**Fig. 4:**
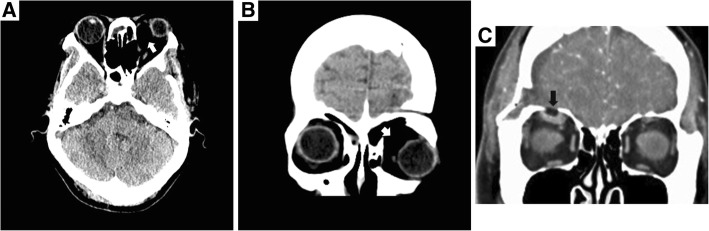
CT findings of bone wax in orbit in our case (4-**a**,4-**b** white arrows) is consistent with hypodense or fat density lesions as described in previous paper [5] (4-**c** black arrow)

Complications related to bone wax migration are rare, and the exact incidence is unknown [1]. To our knowledge, from 1995 to 2018, 19 patients reported complications related to bone wax migration (Table 1) [1, 5–11]. The majority of these cases (14/19) are related to bone wax migration into the sigmoid sinus [7–9], and only one case presented bone wax migration that resulted in ophthalmic symptoms in the orbit [5]. Most of the symptoms were caused by bone wax compression and were partially or completely relieved after surgical removal. As in our case, the CT images of bone wax usually reveal hypodense or fat density lesions (Fig. 4) [5]. However, there is no literature examining MRIs of bone wax in the orbit. In our case, both T1-weighted and T2-weighted MRIs showed a homogeneous hypointense but sharply marginated mass compared with adjacent tissue, with no enhancement (Fig. 2-c).

**Table 1:** Summary of bone wax migration patients reported in the literature from 1995 to 2018

Article	Study Type	Surgery	Bone Wax Migration	Number
Hadeishi et al. 1995 [7]	Retrospective review	Retromastoid craniectomy	Sigmoid sinus	7
Crocker et al. 2008 [8]	Clinical report	Retromastoid craniectomy	Sigmoid sinus	1
Kumar et al. 2009 [6]	Clinical report	Thoracotomy	Epidural migration through spinal canal	1
Byrns et al. 2016 [9]	Retrospective review	Posterior fossa	Sigmoid sinus	6
Spennato et al. 2016 [10]	Clinical report	Craniotomy	Left frontal horn of the lateral ventricle	1
Baird et al. 2018 [11]	Clinical series	Mastoidectomy	Postauricular wound	2
Maki et al. 2017 [5]	Clinical report	Craniotomy	Orbit	1

**Fig. 5:**
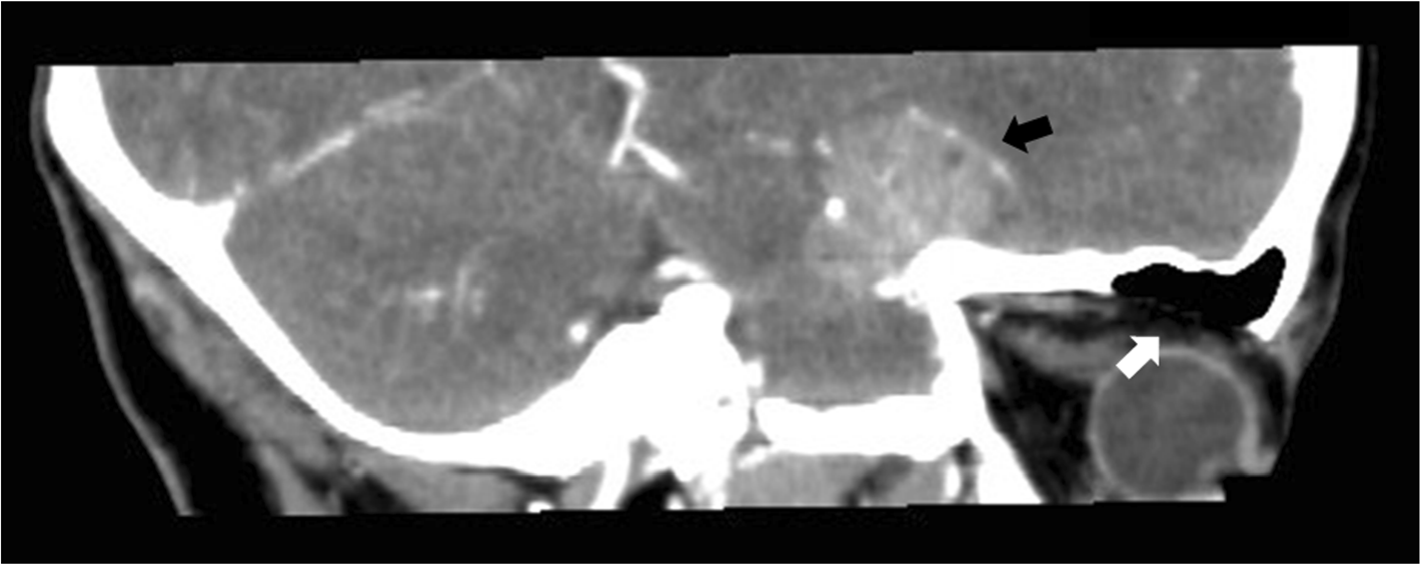
Preoperative brain CT: a bone defect has been found in the basal frontal sinus (white arrow), which might be the pathway the bone wax migrated to the orbit. Black arrow: the mass in sellar-suparsellar-parasellar region before the craniotomy

Regarding the postoperative ptosis after a craniotomy, a postsurgical downward shift of the brain due to gravity might have contributed to the tight packing of the bone wax in the orbit [5]. Additionally, a mismatch between the size of bone wax and the bone window may have caused the displacement of bone wax [5]. Moreover, bone defects in the basal frontal sinus were found in the preoperative CT (Fig. 5). Because the patient had no history of head surgery or trauma, we considered her basal frontal sinus to have a congenital defect. The frontal sinus has substantial diversity [12] in general, with shaped triangles that are sometimes irregular and asymmetrical. The bottom wall of the frontal sinus forms the superior orbital wall, which is very thin. In the absence of bone structure on the bottom of the frontal sinus, the brain tissue and/or cerebrospinal fluid squeezes the bone wax, which is filled in the bone window under the action of gravity, and pushes it into the frontal sinus. Thus, the bone wax is allowed to pass through the enlarged basal frontal sinus and migrate into the orbit, causing compression to the eyeball as well as adjacent tissues.

Complications related to bone wax migration are mostly related to the fact that it is a nonabsorbable agent and remains in the body indefinitely. Therefore, removal of bone wax after a certain period of time should be considered if it is feasible to do so [1]. Additionally, a comprehensive understanding of the patient’s condition and of the structure surrounding the lesion before surgery is also an important prerequisite for preventing postoperative complications.

## Conclusions

We reported a case of craniotomy in which postoperative ptosis, proptosis and defect vision field were observed. These symptoms were considered to be related to the intraoperative application of bone wax. Furthermore, defects in the bone structure of the frontal sinus are also a possible reason for the migration of bone wax. Our case is the first and important example that describes the rare complications caused by migrated bone wax combined with a frontal sinus abnormality. These findings may indicate that extra caution should be taken with the application of bone wax, and a comprehensive understanding of the patient’s intracranial anatomy is important for decreasing the incidence of bone wax migration. Additionally, when a patient presents with symptoms of ocular compression, bone wax migration should be considered in addition to typical radiological changes.

## Abbreviations

BCVA: Best corrected visual acuity
CT: Computed tomography
IOP: Intraocular pressure
mo.: Month
MRI: Magnetic resonance imaging
POD: Postoperative day
